# The effects of viral load on pseudorabies virus gene expression

**DOI:** 10.1186/1471-2180-10-311

**Published:** 2010-12-06

**Authors:** Judit S Tóth, Dóra Tombácz, Irma F Takács, Zsolt Boldogkői

**Affiliations:** 1 Department of Medical Biology, Faculty of Medicine, University of Szeged, Somogyi B. st. 4., Szeged, H-6720, Hungary

## Abstract

**Background:**

Herpesvirus genes are classified into distinct kinetic groups on the basis of their expression dynamics during lytic growth of the virus in cultured cells at a high, typically 10 plaque-forming units/cell multiplicity of infection (MOI). It has been shown that both the host response and the success of a pathogen are dependent on the quantity of particles infecting an organism. This work is a continuation of an earlier study [1], in which we characterized the overall expression of PRV genes following low-MOI infection. In the present study, we have addressed the question of whether viral gene expressions are dependent on the multiplicity of infection by comparing gene expressions under low and high-MOI conditions.

**Results:**

In the present study, using a real-time RT-PCR assay, we address the question of whether the expression properties of the pseudorabies virus (PRV) genes are dependent on the number of virion particles infecting a single cell in a culture. Our analysis revealed a significant dependence of the gene expression on the MOI in most of these genes. Specifically, we found that most of the examined viral genes were expressed at a lower level at a low MOI (0.1) than at a high MOI (10) experiment in the early stage of infection; however, this trend reversed by six hour post-infection in more than half of the genes. Furthermore, in the high-MOI infection, several PRV genes substantially declined within the 4 to 6-h infection period, which was not the case in the low-MOI infection. In the low-MOI infection, the level of antisense transcript (AST), transcribed from the antiparallel DNA strand of the immediate-early 180 (*ie180*) gene, was comparable to that of *ie180 *mRNA, while in the high-MOI experiment (despite the 10 times higher copy number of the viral genome in the infected cells) the amount of AST dropped by more than two log values at the early phase of infection. Furthermore, our analysis suggests that adjacent PRV genes are under a common regulation. This is the first report on the effect of the multiplicity of infection on genome-wide gene expression of large DNA viruses, including herpesviruses.

**Conclusion:**

Our results show a strong dependence of the global expression of PRV genes on the MOI. Furthermore, our data indicate a strong interrelation between the expressions of *ie180 *mRNA and AST, which determines the expression properties of the herpesvirus genome and possibly the replication strategy (lytic or latent infection) of the virus in certain cell types.

## Background

Pseudorabies virus (PRV), an alpha-herpesvirus, and the causative agent of Aujeszky's diseases of swine [2], is a commonly used model organism for studies in pathogenesis and the molecular biology of herpesviruses. Furthermore, it is widely utilized as a neural circuit tracer [[3,4] and [5]] and has been reported to be suitable as a vector for gene delivery to various cells [6,7] and as an oncolytic agent [8]. The gene expressions of herpesviruses are currently undergoing intensive investigation in consequence of the development of new technologies allowing simultaneous analysis of the expressions of multiple genes. DNA microarray approaches have been applied for the overall analysis of herpesvirus gene expression in several studies [[9,10] and [11]]. Microchip techniques are powerful tools that permit simultaneous measurement of the relative changes in quantity of thousands of genes of an organism, and the comparison of gene expression profiles under various circumstances. Quantitative real-time RT-PCR is a much more sensitive and accurate method, but, at least at present, it is not well suited for the analysis of large numbers of samples. The herpesvirus genome however is, within the range that can be successfully analysed with this technique [1].

The program of herpesvirus gene expression is controlled at multiple levels by complex interactions between viral and cellular factors. The lytic gene expressions of herpesviruses are strictly coordinated in a sequential cascade manner and are traditionally subdivided into immediate-early (IE), early (E) and late (L) phases. IE proteins are involved in the control of the synthesis of E and L genes. The IE180 gene (*ie180*; homologue of the HSV ICP4 gene) is the only IE gene of PRV, and the most important regulator of viral gene expression. The E genes of herpesviruses are involved in various aspects of DNA synthesis, while most L genes mainly encode the structural elements of the virus. The antisense transcripts LLT (long latency transcript) and LAT (latency-associated transcript) overlapping the ICP4 and ICP0 (a homologue of *ep0 *in PRV), respectively, are reported to play important roles in the establishment of latency in HSV [12]. It has not yet been unequivocally clarified whether the expression of antisense transcript produced by the complementary DNA strand of the *ie180 *gene is controlled solely by the LAP (LAT promoter) producing LLT or also by a putative promoter (antisense promoter, ASP) localized on the inverted repeat of the PRV genome, producing a shorter transcript. In this study, we use the term 'antisense transcript' (AST) for the RNA molecule transcribed from the complementary DNA strand of the *ie180 *gene.

It is well known that both the host response and the success of a pathogen are dependent on the quantity of particles infecting an organism; and, specifically in herpesviruses, the infecting dose determines whether the virus enters a latent state or induces an acute infection [13]. A further important question is whether the global gene expression profile of the virus genome is dependent on the number of virus particles entering the cells. In both traditional and microarray studies, herpesvirus gene expression has been analysed by using a relatively high multiplicity of infection, typically MOI~10 plaque-forming unit (pfu)/cell [9-11]. Theoretically, it is possible that herpesviruses express their genomes in a different manner when only a single virus particle infects a cell as compared with the situation when multiple virions enter a cell. In the present study, we addressed this issue by using low (0.1 pfu/cell) and high (10 pfu/cell) MOIs for the infection of cultured porcine kidney epithelial cells with wild-type PRV, and subsequently analysed and compared the expressions of 37 PRV genes and two antisense transcripts (AST and LAT) using the SYBR Green-based real-time RT-PCR technique.

## Results and Discussion

### Experimental design

**Figure 1:**
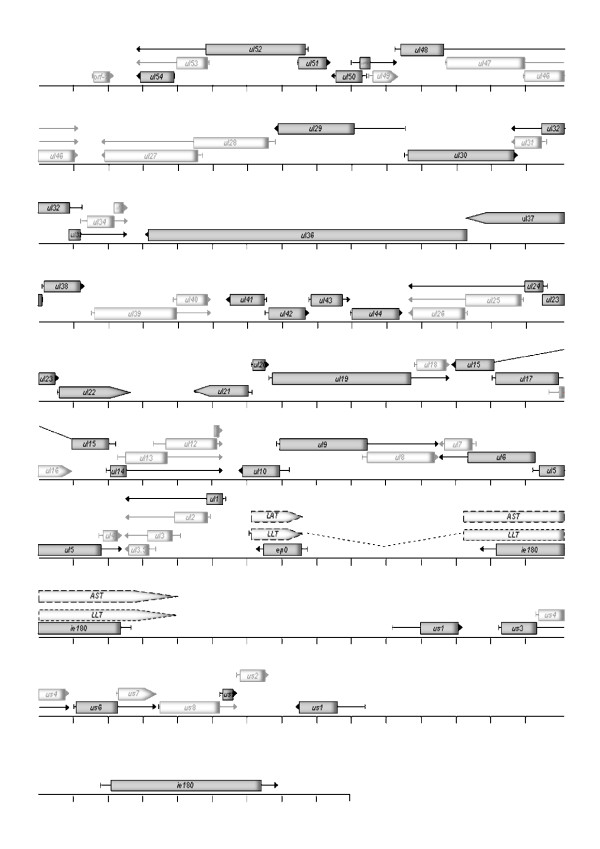
**Localization of PRV genes on the viral genome**. This Figure shows the genomic locations of the PRV genes. The direction of transcription is indicated by the arrows. Grey boxes indicate examined genes. Broken-line boxes show the known antisense transcripts of PRV. Unexamined genes are shown as white boxes.

**Figure 2:**
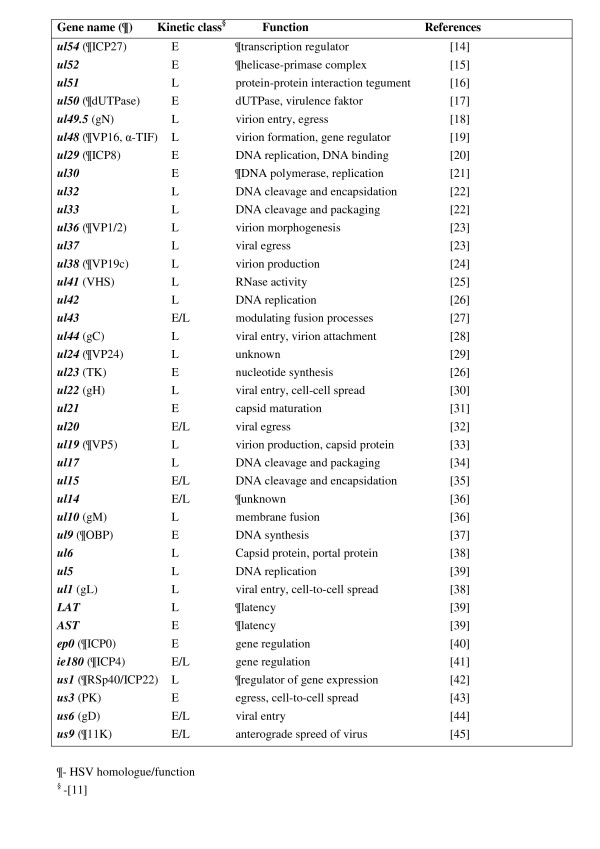
**List of PRV genes analysed in this study**. This Figure presents the kinetic classification of the examined PRV genes, and their functional assignment.

In this study, PK-15 cells were infected with pseudorabies virus at MOIs of 0.1 and 10. Albeit the difference in the infectious dose in the two parallel experiments was 100-fold, an individual cell was invaded by only 10 times more virus particles in the high-MOI than in the low-MOI experiment (5 × 10^6 ^versus 5 × 10^5 ^infected cells), the reason for this being that in the latter case approximately 90% of the cells remained uninfected. Cells were harvested at 0, 1, 2, 4 and 6 h post-infection (pi), as in our earlier report [1]. We used 6 h as the maximum infection period in order to exclude the possibility of the initiation of new infection cycles in the low-MOI experiment. In this study, we analysed the expression of 37 genes (53% of the total PRV genes) and two antisense transcripts (AST and LAT) (Figure 1 and 2[14-45]). For the calculation of relative expression ratios (Rs) (Additional file 1a), we used the average 6 h E^Ct ^values of the high-MOI experiments of both the "samples" and the "references" as controls, as in our earlier publication [1]. We used a correction factor of 10 for the calculation of R for the low-MOI experiment (Additional file 2a). With this calculation technique, approximately the same numbers of infected cells, and hence the relative amounts of transcripts in an average infected cell, were compared in the two experiments. However, in the high-MOI experiment, the proportion of the genome copy number in an infected cell was also 10-fold higher on average, at least before the start of viral DNA replication (the first 2 h pi), the reason for this being that in the high-MOI experiment 10 virus particles infected an average cell, while in the low-MOI infection 10 per cent of the cells were infected with a single virus particle. Thus, to compare the gene expressions from a single virus DNA per cell, two normalizations are necessary: multiplication of the R values of the low-MOI data by 10, and division of the R values of the high-MOI data by 10. In some calculations, the original data were handled accordingly (see the indications in the particular cases). The relationship between the infectious dose and the genome copy number of PRV becomes non-linear in later stages of viral infection; the DNA copy numbers in the two experimental situations are therefore not comparable on the basis of the infectious dose. The R values of LAT and AST were calculated by using the 6 h E^Ct ^values of the corresponding genes, *ep0 *and *ie180*, respectively, as the reference gene. R_Δ _values were used to monitor the net change in the quantity of viral transcripts within a given period of time (Additional file 2b). R_a _shows the ratio of the changes in the amounts of transcripts between two adjacent time points (Additional file 2c).

We considered two principles for the selection of genes for expression analyses. (1) We analysed the upstream genes of each nested gene cluster, the reason for this being that these genes are not overlapped by other genes, and the amounts of these transcripts are therefore proportional to their protein products. This is in contrast with the downstream genes, which, if transcribed from the promoter of an upstream gene, are not translated, because they do not have cap sequences that are required for the recognition by the ribosomes. (2) Furthermore, we analysed genes that are of primary importance in the regulation of global viral gene expression, such as *ie180*, *ep0*, *vhs *and *ul54*.

### Gene expressions in the early stage of PRV infection

In the first 2 h of infection, the viral DNA replication has not yet been initiated, and the copy number of viral genomes in a cell therefore corresponds with the infectious dose. In this analysis, we found that the mRNA levels of most examined PRV genes were higher in the cells infected with the high MOI than in those infected with the low MOI (Additional file 2a) at both 1 h and 2 h pi. This was not unexpected since in the former case viral DNAs were represented in an approximately 10-fold higher proportion in an average infected cell. Exceptions to this were the transcripts *ul1*, *ul33*, and *ul51 *mRNAs at 1 h pi, and *ul36, ul38, ul43*, and *ul48 *mRNAs at 2 h pi, and at both 1 h and 2 h: *ie180 *and *ul30 *mRNAs, as well as, LAT and AST. However, the expression levels normalized to the genome copy number (i.e. using R/10 values in the high-MOI infection) showed an inverse pattern: only a few genes were expressed at higher abundance in the high-MOI than in low-MOI infection (Additional file 2a). AST was expressed at a considerably higher quantity in the cells infected with the low MOI than in those infected with the high MOI (R_low MOI_/R_high MOI _= 111-fold at 1 h, and 298-fold at 2 h pi). The expression rate of a single genomic region encoding the AST was even 10 times higher (1 h: 1110-fold and 2 h: 2980-fold) in the low-dose infection experiment (Additional file 2a). In the high-dose infection 6 of the 37 genes (*ie180*, *ul36*, *ul50*, *ul54*, *us1*, and *ul24*) exhibited higher expression levels at 1 h than at 2 h pi. It should be noted that 3 of them (*ie180*, *us1 *and *ul54*) are regulatory genes. The fourth regulatory PRV gene, *ep0*, is expressed at a very high level during the first 2 h in the high-MOI infection (R_1 h _= 1.87, R_2 h _= 2.05). Apart from *ep0*, *ul5 *(R_2 h _= 1.2) was the only gene that was expressed at a higher extent in the early stages of infection than at 6 h pi in the high-MOI experiment. The *ie180 *gene is the only one that was expressed in a higher amount at 1 h than at 2 h pi under both experimental conditions (Additional file 2). Overall, it appears that the 4 regulatory genes were expressed at relatively high levels before the onset of DNA replication in the high-MOI infection, which was not the case in low-MOI infection, with the exception of the *ie180 *gene. We think that the reason for the higher expression of regulatory genes at the onset of viral DNA replication in the high-MOI infection is that more regulatory proteins are needed to carry out the multiplication of a higher copy number of the viral genome. The rate of change in gene expression within the 1 h to 2 h interval (R_2h_/R_1h_) was higher in more than two-thirds of the PRV genes (25/37) in the low-MOI than in the high-MOI infection (Additional file 2c). The proportion of AST to *ie180 *mRNA molecules (R_AST_/R_ie180_) was 0.47 at 1 h pi, and 4.72 at 2 h pi in cells infected with the low MOI, while this ratio was extremely low (~0.01) at both 1 h and 2 h pi in the high-MOI infection (these data are only semi-quantitative since the primer efficiencies in the RT reaction are not necessarily equal for the two transcripts). Thus, the proportion of AST to *ie180 *mRNA [(R_AST-low MOI_/R_ie-low MOI_)/(R_AST-high MOI_)/R_ie-high MOI_)] was 39-fold higher at 1 h pi and 293-fold higher at 2 h pi in the low-MOI than in the high-MOI infection. In the early stages of PRV infection, the amount of AST was very high; it even significantly exceeded the level of *ie180 *mRNAs at 2 h pi in the low-MOI infection, while the amount of AST and also its ratio to *ie180 *mRNA were extremely low in the high-MOI infection. Moreover, *ie180 *mRNA is expressed to a significantly higher extent in the low-MOI experiment despite the 10 times lower copy number of PRV DNA in an infected cell, which is especially important because IE180 is a DNA-binding protein. We think that this observation reveals an important regulatory mechanism of the herpesviruses, which is as follows: in a high-titre infection, the virus initiates a lytic infection in a cell, while in a low-titre infection, the virus has the choice of whether to establish a dormant state or enter a lytic cycle in a cell. The molecular mechanism of this phenomenon might be based on the interaction of *ie180 *and AST genes at both the transcription and translation levels. (1) The ie180 protein might exert a negative effect on the synthesis of AST, such as in LAT in HSV [46] by binding the promoter of the antisense transcript. (2) Furthermore, the complementary transcripts might mutually influence each other's expression transcript by RNA-RNA interaction. In a low-MOI infection, the two transcripts exhibit a complementary expression pattern, which indicates a competition between the two transcripts. In a high-MOI infection, however, the high initial amount of *ie180 *gene product inhibits the expression of AST. The significance of this infection strategy could be that, in the case of a low-amount infection, the virus has no chance to invade the host cells; therefore, it is better to hide against the immune surveillance.

The *ep0 *gene is expressed in higher quantity at both 1 h pi (4.22-fold) and 2 h pi (2.43-fold) in the high-MOI infection than in low-MOI infection, which is in contrast with LAT, its antisense partner, whose expression level was lower in the high-MOI infection (1 h: 0,5-fold; 2 h: 0,18-fold). Thus, the ratios of LAT to *ep0 *mRNA molecules were 8.33-fold higher at 1 h pi and 13.05-fold higher at 2 h pi in the low-MOI than in the high-MOI experiment, although, unlike AST, LAT is abundantly expressed in the high-MOI infection. Accordingly, similarly to AST, LAT is expressed in a significantly higher proportion to *ep0 *mRNA in the low-MOI infection in the early stages of infection, which may also be important as concerns of the replication strategy of the virus. Our analyses additionally showed that AST and LAT are, at least partly, expressed independently from each other, which supports the existence of separate elements controlling the expressions of the two antisense transcripts. Indeed, AST was suggested to be controlled by an antisense promoter (ASP) localized in the outer regions of inverted repeats [47].

### Gene expressions in later stages of PRV infection

At 4 h pi the transcript levels of more than three-quarters of the PRV genes (28/37) were still higher in the cells infected with the high MOI than in those infected with the low MOI (Additional file 2c). However, in about two-third of the viral genes the rate of change (R_a _values) in the expression level was higher in the low-MOI than in the high-MOI infection (24/37 within the 2 h to 4 h period, and 25/37 within the 1 h to 4 h period) (Additional file 2c). In the low-MOI infection, the amounts of 5 transcripts (*ul5*, *ul44*, *us1 *and *us6*) were less than 10% of those in the high-MOI infection at 4 h pi. All of the examined *us *genes are expressed at a significantly lower level in the low-than in the high-titre infection at 4 h pi. There were significant decreases in the quantities of both AST and LAT in the low-titre infection at 4 h pi relative to the 2 h values (AST: a 59-fold decrease, and LAT: a 7-fold decrease). We explain this phenomenon by the negative effect of the regulatory genes on their antisense partners. Regulatory genes are upregulated at the onset of DNA replication (in order to facilitate this process), which exerts an inhibitory effect on the expression of AST and LAT. In contrast, there were increases in the amounts of antisense transcripts in the high-MOI (AST: an 11-fold increase, and LAT: a 7-fold increase) in this time interval. However, while LAT was expressed at high level (R = 1.3) under the high-MOI conditions, the AST expression remained extremely low (R = 0.013) in this period of infection. The amount of the *ie180 *transcript was practically unchanged within the 2 h to 4 h infection period under either infection conditions. There was a 4.7-fold increase in the *ep0 *mRNA level within the 2 h to 4 h infection period (R_4h_/R_2h_) in the low-MOI infection, as compared with only 1.4 in the high-MOI experiment. On average, the amounts of mRNAs in low titre infection became higher than those in the high-infection titre by 6 h pi in more than half of the PRV genes (22/37). We assume that the reason for this might be that the *ie180 *gene, the major coordinator of gene expression, is expressed at higher levels at 4 and 6 h pi at low-MOI than at high-MOI infection. Moreover, in the high-MOI infection the amount of AST reached almost 30% of the transcript level in the low-MOI infection, while LAT was expressed at approximately the same level under the two infection conditions at 6 h pi. The genes expressed at lower levels in the low-dose infection appeared to be clustered on adjacent genomic locations (Figure 1). Each gene and the two antisense transcripts were expressed at higher rates (R_a _values) within the 4 h to 6 h period in the low-MOI than in the high-MOI infection without exception (Additional file 1c**)**. In the high-MOI infection, 11 genes and LAT peaked at 4 h within the 6-h examination period, while in the low-MOI infection only the *us3 *transcript had a slightly lower R value at 6 h than at 4 h pi. The *us3 *gene was the only one among the 70 PRV genes which was expressed at a higher level at 4 h than at 6 h pi in another study [1]. Intriguingly, the *ep0 *mRNAs reached a 3.5-fold higher level in the low-dose than in the high-dose infection in an average cell at 6 h pi. Furthermore, at 6 h pi the *ul1 *and *ul51 *genes were expressed at an approximately 10 times higher level under the low-MOI than under the high-MOI conditions.

### Gene expression kinetics within the 0 to 6-h infection period

**Figure 3:**
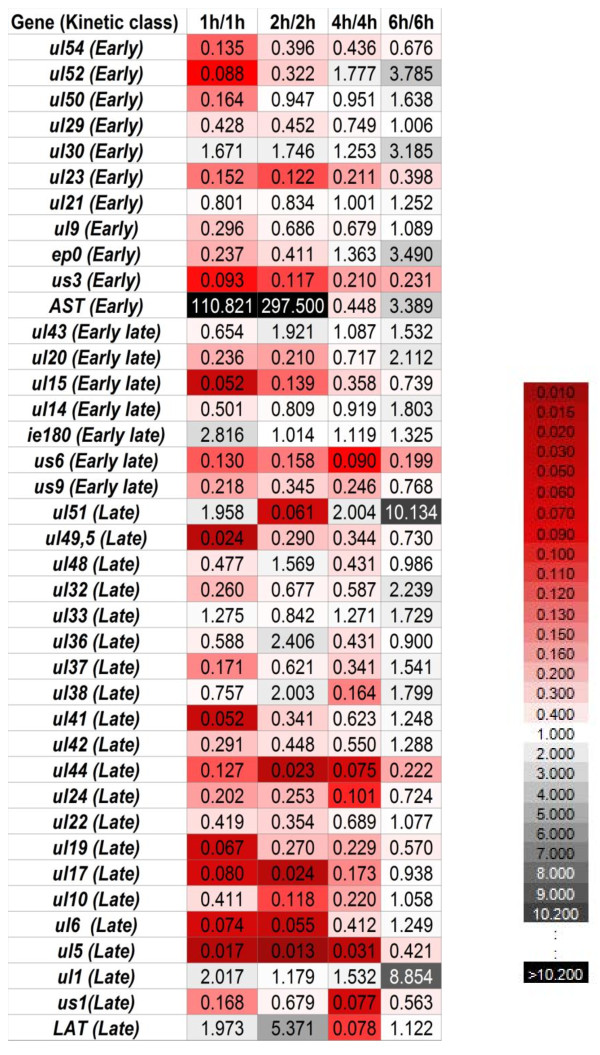
**Heatmap-like representation of the ratio of transcripts produced in the low-MOI and high MOI infection (R_t low MOI/_R_t high MOI_)**. PK-15 cells were infected with the PRV-Ka strain at different MOIs (0.1 and 10). Real-Time PCR data were normalised to 28 S RNAs. The R_low_/R_high _values are plotted in a heat map-like manner. Black boxes indicate the highest ratio, and dark-red boxes the lowest values. White boxes demonstrate approximately equal values.

**Figure 4:**
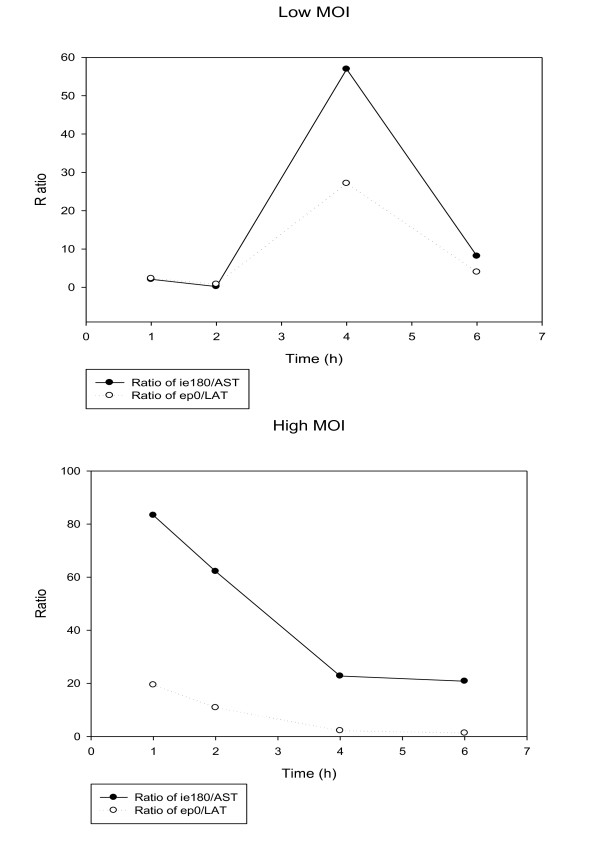
**The ratio of *ie180 *and *ep0 *mRNAs to their antisense partners**. The continuous lines illustrate the ratio of *ie180 *mRNA to AST, while the dotted lines represent the ratio of *ep0 *mRNA to LAT at the low- and high-MOI infections.

**Figure 5:**
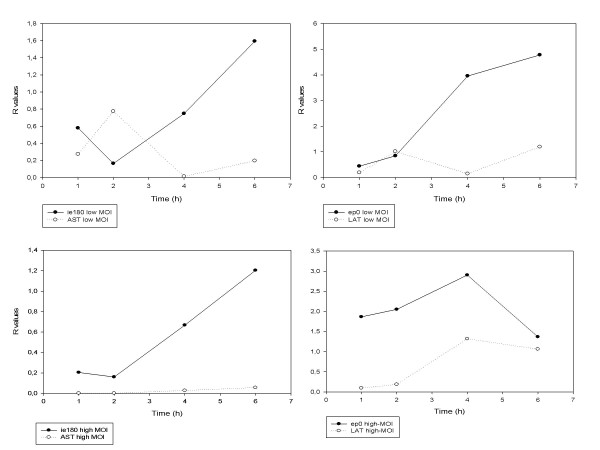
**The R values of *ie180 *and *ep0 *mRNAs and their antisense partners**. These diagrams depict the expression curves of sense and antisense transcripts of two regulatory genes (*ie180 *and *ep0*) at the different infectious doses. The continuous lines represent the level of sense transcripts at the given time points, while broken lines show the amounts of their antisense counterparts.

The expression of most PRV genes basically differed under the two infection conditions (Additional file 1c), which is in contrast with the case of rhesus monkey rhadinovirus (a γ-herpesvirus), whose lytic gene expression commences at a fixed pace in infected cells, regardless of the MOI [48]. Most genes were expressed at a lower level in a cell in the low-MOI experiment in the first 4 h of infection, but more than half of these gene products surpassed the high-MOI values by 6 h pi. The R values of 3 PRV genes (*ie180, ul1 and ul30*) were higher in the low-MOI than in the high-MOI infection at every examined time point, while the opposite was true (the R values of high-MOI were always higher) in 13 genes: *ul5, ul15, ul17, ul19, ul23, ul24, ul44, ul49.5, ul54, us6, us9, us1 and us3 *(Figure 3). These latter genes form clusters on the basis of their localization on the genome (genes in close vicinity are underlined), which suggests that the adjacent genomic sequences might be under common regulatory control. This observation is supported by the similarity of the R_a _curves of adjacent genes (Additional file 1c). For example, the expression rates of the *ul36*, *ul37 *and *ul38 *genes were similar to each other in both experiments, but each of them exhibited an inverse expression pattern in the two infection conditions. All genes were expressed at a higher rate (R_a_) within the 1 h to 6 h period of infection in the low-titre experiment, except for *ie180 *and the two antisense transcripts. The quantities of *ie180 *mRNAs were similar in the two experiments, except at 1 h pi, where the level of the transcripts was 2.8-fold higher in the low-MOI infection. Thus, the amount of total *ie180 *transcript in an infected cell appears to be under strict control, independently of the initial infection conditions. In contrast, the expression of the *ep0 *gene differed basically in the two experiments. In the high-MOI experiment, the amount of *ep0 *mRNAs was high from the first hour of infection, and its expression even declined by 6 h in the high-MOI infection, while the amount of these transcripts rapidly increased throughout the 6-h infection period in the low-dose infection, and reached a 3.5-fold higher level compared to that of the high-dose infection by 6 h. (Figure 4) The ratio of sense and antisense transcripts during the 6-h infection period displayed intriguing patterns. First of all, in the high-MOI infection the amount of AST and its ratio to *ie180 *mRNA were very low throughout the 6-h infection period. We demonstrated an inverse relationship in the expression kinetics of *ie180 *mRNA and AST and also *ep0 *mRNA and LAT in the low-MOI infection; however, we did not observe this inverse relationship between the complementary transcripts under the high-MOI conditions (Figure 5). In an earlier report [1], we showed that treatment of infected cells with cycloheximide (a protein synthesis blocker) resulted in significant increases in the amounts of both *ie180 *mRNA and AST, while phosphonoacetic acid (a DNA synthesis inhibitor) treatment led to a decrease in *ie180 *mRNA and a significant increase in the AST level. These results suggest a negative effect of the IE180 transactivator on ASP synthesis. We explain the huge drop in ASP level in the infected cells in the early stage of the high-MOI infection by the presence of a 10-fold higher amount of inhibitory IE180 protein localized in the tegument of the infecting virions [49]. The same reason could account for the lower *ie180 *mRNA level in the high-MOI infection. The *us1 *gene was expressed in the late kinetics in our earlier low-MOI analysis in both phophonoacetic acid-treated and non-treated samples. These results are in concordance with those of the present high-dose infection experiment, i.e. *us1 *mRNA was expressed at a relatively low level at 1 h, which even dropped by 2 h pi. The highest rate of *us1 *mRNA expression was observed at 4 h, with a rate (R_4 h/2 h _= 13.32) typical of L genes. The Pearson correlation coefficients of the R, R_Δ_, and R_a _values precisely show the degree of similarity (or differences) of the expression kinetics of the genes in the low- and high-MOI experiments (Additional file 3). Several genes exhibited high correlations for all three parameters. For example, the *ie180*, *ul19*, *ul21*, *ul22*, *ul42 *and *ul43 *genes gave high correlation coefficients for the R, R_Δ _and R_a _values. The *us1 *gene behaved in an irregular manner; it gave a relatively high correlation for the R values, no correlation of R_Δ_, and an inverse correlation for the R_a _values. AST yielded relatively high negative values for all three parameters, indicating a significant negative correlation. The expressions of LAT under the two experimental conditions did not correlate on the basis of the R values, whereas it gave a very high negative correlation for its R_Δ _and R_a _values. The effect of the MOI on the overall gene expression of HSV-1 has been investigated by Wagner and colleagues [50], who found that, following the infection of cultured cells by wild-type virus at MOIs ranging from 0.05 to 5 pfu/cell, the temporal profiles of transcript abundance were essentially the same. This is in sharp contrast with our results. We explain the differences by the low resolution of the microarray technique that Wagner et al. used for their analysis. An analysis of the global transcription of Rhesus monkey rhadinovirus, a γ-herpesvirus, has revealed differential gene expression at different MOIs [48], but these data cannot be compared because they related to later time points (12, 24, 48 72 and 96 h) than in our analysis.

## Conclusion

Our analysis has revealed that almost all of the examined PRV genes exhibited different expression dynamics under the two experimental conditions. Most PRV genes were expressed at a lower level in the low-MOI than in the high-MOI experiment in the early stages of infection; however, the reverse was true when the transcript levels were normalized to the genome copy numbers. In the low-MOI infection, slightly more than half of the PRV transcripts outran the high-MOI values by 6 h pi. The lower *ie180 *transcript per genome in the high-titre infection experiment might account for the lower level of global PRV gene expression per genome in the high-MOI infection. However, the expression of viral genes per DNA did not uniformly decrease; some genes even became more active in the high-MOI infection, which indicates the selective effect of the reduced availability of the IE180 protein. The most dramatic difference between the two MOI infections was observed in AST, which was expressed at a more than two log higher level in an infected cell in the low-MOI infection, which is a 3 log higher activity of a single DNA region encoding the ASP. The ratio of LAT/EP0 was also significantly lower in the high-than in the low-MOI infection. The reasons for and the mechanisms of these phenomena remain to be clarified. Furthermore, genes localized in adjacent regions on the viral genome exhibited similar expression properties, indicating the existence of synchronizing mechanisms of gene expression.

## Methods

### Virus, cells and infection

Strain Kaplan (Ka) of pseudorabies virus (PRV) was used in our analyses. Immortalized porcine kidney (PK)-15 epithelial cells were applied for propagation of the virus. PK-15 cells were cultivated in Dulbecco's modified Eagle medium supplemented with 5% foetal bovine serum (Gibco Invitrogen) and 80 *μ*g gentamycin per ml at 37°C in the presence of CO_2_. The virus stock used for the experiments was prepared as follows. Rapidly-growing semi-confluent PK-15 epithelial cells were infected at an MOI of 0.1 pfu/cell and were incubated until a complete cytopathic effect was observed. The cell debris was removed by low-speed centrifugation (10,000 g for 20 min). The supernatant was concentrated and further purified by ultracentrifugation through a 30% sugar cushion at 24,000 rpm for 1 h, using a Sorvall AH-628 rotor. The number of cells in a culture flask (Corning, 150 cm^2^) was 5 × 10^6^. In high-MOI and in low-MOI experiments, 5 × 10^7 ^and 5 × 10^5 ^pfu viral particles, respectively, were applied for the infections. Thus, in the high-MOI experiment, practically all the cells were infected, while in the low-MOI experiment, approximately 5 × 10^5 ^cells (10% of the cells in a culture flask) were infected by the virus. We used the same data for the low-MOI experiment as in a previous publication [1]. The two experiments were run simultaneously. We ran four independent sets of measurements for each time point in both low and high-MOI studies, but occasionally we had to remove data because of low amplification efficiencies or the amplification of non-specific products in the reaction." Thus, in some genes, instead of four, we only used three independent data. Infected cells were incubated for 1 h, followed by removal of the virus suspension and washing with phosphate-buffered saline. After the addition of new medium to the cells, they were incubated for 0, 1, 2, 4 or 6 h. In this study, mock-infected cells were used as controls, which were otherwise treated in the same way as the infected cells.

### Isolation of RNAs

RNA was extracted by using the NucleoSpin RNA II Kit (Macherey-Nagel GmbH and Co. KG), as described previously [1]. Briefly, after the cells had been collected by centrifugation and lysed by buffer containing chaotropic ions, the nucleic acids were docked to a silica column. The DNA was removed with RNase-free DNase solution (supplied with the NucleoSpin RNA II Kit). Finally, the RNAs were eluted from the column in RNase-free water (supplied with the kit). To eliminate the residual DNA contamination, all RNA samples were treated by an additional digestion with Turbo DNase (Ambion Inc.). The concentrations of the RNA samples were measured by spectrophotometric analysis with a BioPhotometer Plus instrument (Eppendorf). RNA samples were stored at -80°C until further use.

### Reverse transcription

0.07 *μ*g of total RNA was reverse transcribed in a 5 *μ*l reaction volume, using SuperScript III Reverse Transcriptase (Invitrogen), a gene-specific primer (1 *μ*l), dNTP mix (0.25 *μ*l; 10 *μ*M final concentration), and 5× First-Strand Buffer (1 *μ*l). The reaction mix was incubated at 55°C for 60 min and incubation was stopped by holding at 70°C for 15 min. A no-RT control reaction was run to ensure that the RNA samples were free of DNA contamination. For the quantitative RT-PCR reactions, only DNA-free RNA samples were used. First-strand cDNAs were diluted 10-fold with Nuclease-Free Water (Promega Corp.) and stored at -80°C until use. The same primers were used for the RT reaction as in our previous publication [1].

### Real-time PCR

A Rotor-Gene 6000 cycler (Corbett Life Science) was used for the real-time quantitative PCR analysis. Each reaction (20 *μ*l final volume) contained the following components: 7 *μ*l of cDNAs, 10 *μ*l of Absolute QPCR SYBR Green Mix (Thermo Fisher Scientific), 1.5 *μ*l of forward and reverse primer (10 *μ*M each; we used the same primer pairs as described earlier [1]). The PCR cycling parameters were as follows: 95°C for 15 min (pre-incubation), and then 30 cycles of 94°C for 25 sec (denaturation), 60°C for 25 sec (annealing), and 72°C for 6 sec (extension). The specific amplification products (with a single peak at the predicted temperature) were identified by melting-point curve analysis. An additional detection step was included in the cycle program to avoid primer dimer detection for those primer pairs that produce primer dimers. The reliability of the primers was verified in our earlier publication [1]. Porcine 28 S rRNA was used as a loading control throughout the experiment. H_2_O was included as a no-template control, and cDNA derived from the reverse-transcribed RNAs of non-infected cells was used as a negative mock-infected control. SYBR Green-based real-time PCR was applied in this study because of the low costs and simple protocol [51].

### Data analysis

The following formula was used for calculation of the relative expression ratio (R):

R=(Esample6h)Ctsample6h(Esample)Ctsample¯:(Eref6h)Ctref6h(Eref)Ctref¯

where E is the efficiency of amplification, Ct is the cycle threshold value, 'sample' is the examined PRV gene, and 'ref' is the 28 S rRNA. The Comparative Quantitation module of the Rotor-Gene 6000 Software (Version 1.7.87., Corbett Research) was used to calculate the real-time PCR efficiency for each sample. Thresholds were set by the software. The R values of both low and high-titre infections were maximized to the 6 h E^Ct ^values of the high-MOI experiment. To measure the net change in R between two consecutive time points, R_Δ _was calculated via the following formula: R_Δ _= R_(t+1)_-R_t_. The rate of change was calculated as follows: R_a _= R_(t+1)_/R_t_. Pearson's correlation was used for the analysis of the relationship between low- and high-titre infections using the following formula [52]:

$$\text{r}=\frac{\sum_{i=1}^{n}\left(X_{i}-\bar{X})(Y_{i}-\bar{Y}\right)}{(n-1)S_{x}S_{y}}$$

The correlation measures the linear relationship between two variables, X and Y. Pearson's coefficient (r) is a number ranging from -1 to +1 that measures the degree of association between X and Y. If X and Y are independent, Pearson's correlation coefficient is 0. A positive r value for the correlation implies a positive association (large values of X tend to be associated with large values of Y, and small values of X tend to be associated with small values of Y). A negative value for the correlation means an inverse association (large values of X tend to be associated with small values of Y, and vice versa). In the analysis of the relationship between the low and high-titre infections, $\bar{X}$ is the average R value of the low-titre infection at a given time point, and $\bar{Y}$ is the average R value at the same time point in the high-titre infection. S_X _and S_Y _are the SEM (standard error of the mean) values and n is the sample number.

## Competing interests

The authors declare that they have no competing interests.

## Authors' contributions

JT carried out the standard and real-time PCR, the agarose and polyacrilamide gel electrophoresis, and the DNA sequencing, and participated in the evaluation of the primary data. DT took part by performing the reverse transcription reactions, purified PRV RNA, and propagated PK-15 cells. IT participated in performing the reverse transcription reactions. ZB coordinated the study, propagated viruses and isolated viral DNAs. All authors have read and approved the final manuscript.

## Supplementary Material

Additional file 1 **The running curves of R, R_Δ_, and R_a _values**. Click here for file

Additional file 2 **The relative expression ratio (R), the R_Δ_, and R_a _values**. Click here for file

Additional file 3 **Comparison of R, R_Δ _and R_a _values of low and high MOI infection by Pearson correlation**. Click here for file
