# Supplementary material for: The effects of viral load on pseudorabies virus gene expression
Source: BMC Microbiol. 2010 Dec 6;10:311. doi: 10.1186/1471-2180-10-311 (PMC3016322; doi:10.1186/1471-2180-10-311)
Supplement: Additional file 1 — The running curves of R, RΔ, and Ra values. [file 1471-2180-10-311-S1.DOC]

Additional file 1. Gene expression curves

a. R values

Early genes

Early-late genes

Late genes

b. R_∆_ values

Early genes

Early-late genes

Late genes

c. R_a_ values

Early genes

Early-late genes

Late genes
