# Supplementary material for: The effects of viral load on pseudorabies virus gene expression
Source: BMC Microbiol. 2010 Dec 6;10:311. doi: 10.1186/1471-2180-10-311 (PMC3016322; doi:10.1186/1471-2180-10-311)
Supplement: Additional file 2 — The relative expression ratio (R), the RΔ, and Ra values. [file 1471-2180-10-311-S2.DOC]

Additional file 2

**a**. The relative expression ratios (R)

| **High-MOI R values normalized to genome copy number** | | |
| --- | --- | --- |
|  | **1h** | **2h** |
| **ul54** | 0,0299 | 0,0248 |
| **ul52** | 0,0567 | 0,0622 |
| **ul50** | 0,0532 | 0,0354 |
| **ul29** | 0,0401 | 0,0773 |
| **ul30** | 0,0659 | 0,0959 |
| **ul23** | 0,0211 | 0,0478 |
| **ul21** | 0,0077 | 0,0131 |
| **ul9** | 0,0301 | 0,0451 |
| **ep0** | 0,1865 | 0,2050 |
| **us3** | 0,0352 | 0,0695 |
| **AST** | 0,0002 | 0,0003 |
| **ul43** | 0,0118 | 0,0241 |
| **ul20** | 0,0101 | 0,0239 |
| **ul15** | 0,0082 | 0,0228 |
| **ul14** | 0,0153 | 0,0261 |
| **ie180** | 0,0205 | 0,0162 |
| **us6** | 0,0042 | 0,0057 |
| **us9** | 0,0071 | 0,0142 |
| **ul51** | 0,0033 | 0,0833 |
| **ul49,5** | 0,0066 | 0,0084 |
| **ul48** | 0,0063 | 0,0076 |
| **ul32** | 0,0075 | 0,0192 |
| **ul33** | 0,0058 | 0,0075 |
| **ul36** | 0,0291 | 0,0118 |
| **ul37** | 0,0034 | 0,0065 |
| **ul38** | 0,0076 | 0,0125 |
| **ul41** | 0,0207 | 0,0260 |
| **ul42** | 0,0043 | 0,0146 |
| **ul44** | 0,0005 | 0,0018 |
| **ul24** | 0,0028 | 0,0027 |
| **ul22** | 0,0022 | 0,0076 |
| **ul19** | 0,0065 | 0,0083 |
| **ul17** | 0,0024 | 0,0088 |
| **ul10** | 0,0022 | 0,0060 |
| **ul6** | 0,0052 | 0,0817 |
| **ul5** | 0,0241 | 0,1218 |
| **ul1** | 0,0058 | 0,0121 |
| **us1** | 0,0116 | 0,0102 |
| **LAT** | 0,0096 | 0,0188 |

|  | **Low-MOI R values** | | | |  |  | **High-MOI R values** | | | |
| --- | --- | --- | --- | --- | --- | --- | --- | --- | --- | --- |
|  | **1h** | **2h** | **4h** | **6h** |  |  | **1h** | **2h** | **4h** | **6h** |
| **ul54** | 0,04 | 0,10 | 0,39 | 0,68 |  | **ul54** | 0,30 | 0,25 | 0,89 | 1,01 |
| **ul52** | 0,05 | 0,20 | 1,94 | 4,21 |  | **ul52** | 0,57 | 0,62 | 1,09 | 1,11 |
| **ul50** | 0,09 | 0,34 | 1,28 | 1,89 |  | **ul50** | 0,53 | 0,35 | 1,35 | 1,16 |
| **ul29** | 0,17 | 0,35 | 0,96 | 1,08 |  | **ul29** | 0,40 | 0,77 | 1,28 | 1,07 |
| **ul30** | 1,10 | 1,67 | 1,64 | 3,31 |  | **ul30** | 0,66 | 0,96 | 1,31 | 1,04 |
| **ul23** | 0,03 | 0,06 | 0,33 | 0,41 |  | **ul23** | 0,21 | 0,48 | 1,54 | 1,02 |
| **ul21** | 0,06 | 0,11 | 0,88 | 1,37 |  | **ul21** | 0,08 | 0,13 | 0,88 | 1,10 |
| **ul9** | 0,09 | 0,31 | 0,76 | 1,41 |  | **ul9** | 0,30 | 0,45 | 1,12 | 1,29 |
| **ep0** | 0,44 | 0,84 | 3,95 | 4,78 |  | **ep0** | 1,87 | 2,05 | 2,90 | 1,37 |
| **us3** | 0,03 | 0,08 | 0,27 | 0,24 |  | **us3** | 0,35 | 0,70 | 1,30 | 1,03 |
| **AST** | 0,27 | 0,77 | 0,01 | 0,20 |  | **AST** | 0,00 | 0,00 | 0,03 | 0,06 |
| **ul43** | 0,08 | 0,46 | 1,29 | 1,93 |  | **ul43** | 0,12 | 0,24 | 1,19 | 1,26 |
| **ul20** | 0,02 | 0,05 | 0,57 | 2,63 |  | **ul20** | 0,10 | 0,24 | 0,80 | 1,25 |
| **ul15** | 0,00 | 0,03 | 0,25 | 0,77 |  | **ul15** | 0,08 | 0,23 | 0,69 | 1,04 |
| **ul14** | 0,08 | 0,21 | 0,91 | 1,87 |  | **ul14** | 0,15 | 0,26 | 0,99 | 1,04 |
| **ie180** | 0,58 | 0,16 | 0,75 | 1,59 |  | **ie180** | 0,21 | 0,16 | 0,67 | 1,20 |
| **us6** | 0,01 | 0,01 | 0,04 | 0,20 |  | **us6** | 0,04 | 0,06 | 0,48 | 1,02 |
| **us9** | 0,02 | 0,05 | 0,23 | 0,99 |  | **us9** | 0,07 | 0,14 | 0,94 | 1,28 |
| **ul51** | 0,07 | 0,05 | 3,15 | 12,25 |  | **ul51** | 0,03 | 0,83 | 1,57 | 1,21 |
| **ul49,5** | 0,00 | 0,02 | 0,23 | 0,77 |  | **ul49,5** | 0,07 | 0,08 | 0,66 | 1,06 |
| **ul48** | 0,03 | 0,12 | 0,34 | 1,17 |  | **ul48** | 0,06 | 0,08 | 0,79 | 1,18 |
| **ul32** | 0,02 | 0,13 | 1,25 | 3,54 |  | **ul32** | 0,08 | 0,19 | 2,13 | 1,58 |
| **ul33** | 0,07 | 0,06 | 0,70 | 1,92 |  | **ul33** | 0,06 | 0,08 | 0,55 | 1,11 |
| **ul36** | 0,17 | 0,28 | 0,26 | 0,90 |  | **ul36** | 0,29 | 0,12 | 0,59 | 1,00 |
| **ul37** | 0,01 | 0,04 | 0,26 | 1,62 |  | **ul37** | 0,03 | 0,06 | 0,75 | 1,05 |
| **ul38** | 0,06 | 0,25 | 0,18 | 1,91 |  | **ul38** | 0,08 | 0,13 | 1,11 | 1,06 |
| **ul41** | 0,01 | 0,09 | 0,61 | 1,43 |  | **ul41** | 0,21 | 0,26 | 0,97 | 1,15 |
| **ul42** | 0,01 | 0,07 | 0,59 | 1,30 |  | **ul42** | 0,04 | 0,15 | 1,07 | 1,01 |
| **ul44** | 0,00 | 0,00 | 0,04 | 0,24 |  | **ul44** | 0,00 | 0,02 | 0,55 | 1,06 |
| **ul24** | 0,01 | 0,01 | 0,10 | 0,74 |  | **ul24** | 0,03 | 0,03 | 0,95 | 1,03 |
| **ul22** | 0,01 | 0,03 | 0,25 | 1,21 |  | **ul22** | 0,02 | 0,08 | 0,36 | 1,13 |
| **ul19** | 0,00 | 0,02 | 0,19 | 0,80 |  | **ul19** | 0,07 | 0,08 | 0,84 | 1,40 |
| **ul17** | 0,00 | 0,00 | 0,18 | 0,99 |  | **ul17** | 0,02 | 0,09 | 1,06 | 1,06 |
| **ul10** | 0,01 | 0,01 | 0,15 | 1,06 |  | **ul10** | 0,02 | 0,06 | 0,69 | 1,00 |
| **ul6** | 0,00 | 0,04 | 0,35 | 1,50 |  | **ul6** | 0,05 | 0,82 | 0,86 | 1,20 |
| **ul5** | 0,00 | 0,02 | 0,07 | 0,24 |  | **ul5** | 0,24 | 1,22 | 2,19 | 0,56 |
| **ul1** | 0,12 | 0,14 | 1,49 | 10,10 |  | **ul1** | 0,06 | 0,12 | 0,97 | 1,14 |
| **us1** | 0,02 | 0,07 | 0,10 | 0,96 |  | **us1** | 0,12 | 0,10 | 1,36 | 1,71 |
| **LAT** | 0,19 | 1,01 | 0,15 | 1,19 |  | **LAT** | 0,10 | 0,19 | 1,32 | 1,06 |

**b**. The R_∆_ values (R_(t+1)_-R_t_)

|  | **Low MOI** | | | |  |  | **High MOI** | | | |
| --- | --- | --- | --- | --- | --- | --- | --- | --- | --- | --- |
|  | **1h-0h** | **2h-1h** | **4h-2h** | **6h-4h** |  |  | **1h-0h** | **2h-1h** | **4h-2h** | **6h-4h** |
| **ul54** | 0,04 | 0,06 | 0,29 | 0,29 |  | **ul54** | 0,30 | -0,05 | 0,65 | 0,11 |
| **ul52** | 0,05 | 0,15 | 1,74 | 2,27 |  | **ul52** | 0,57 | 0,05 | 0,47 | 0,02 |
| **ul50** | 0,09 | 0,25 | 0,95 | 0,61 |  | **ul50** | 0,53 | -0,18 | 0,99 | -0,19 |
| **ul29** | 0,17 | 0,18 | 0,61 | 0,12 |  | **ul29** | 0,40 | 0,37 | 0,51 | -0,21 |
| **ul30** | 1,10 | 0,57 | -0,04 | 1,67 |  | **ul30** | 0,66 | 0,30 | 0,35 | -0,27 |
| **ul23** | 0,03 | 0,03 | 0,27 | 0,08 |  | **ul23** | 0,21 | 0,27 | 1,06 | -0,52 |
| **ul21** | 0,06 | 0,05 | 0,77 | 0,50 |  | **ul21** | 0,08 | 0,05 | 0,74 | 0,22 |
| **ul9** | 0,09 | 0,22 | 0,45 | 0,64 |  | **ul9** | 0,30 | 0,15 | 0,67 | 0,17 |
| **ep0** | 0,44 | 0,40 | 3,11 | 0,82 |  | **ep0** | 1,87 | 0,19 | 0,85 | -1,53 |
| **us3** | 0,03 | 0,05 | 0,19 | -0,04 |  | **us3** | 0,35 | 0,34 | 0,60 | -0,27 |
| **AST** | 0,27 | 0,50 | -0,76 | 0,18 |  | **AST** | 0,00 | 0,00 | 0,03 | 0,03 |
| **ul43** | 0,08 | 0,39 | 0,83 | 0,64 |  | **ul43** | 0,12 | 0,12 | 0,95 | 0,07 |
| **ul20** | 0,02 | 0,03 | 0,52 | 2,06 |  | **ul20** | 0,10 | 0,14 | 0,56 | 0,45 |
| **ul15** | 0,00 | 0,03 | 0,22 | 0,52 |  | **ul15** | 0,08 | 0,15 | 0,46 | 0,35 |
| **ul14** | 0,08 | 0,13 | 0,70 | 0,96 |  | **ul14** | 0,15 | 0,11 | 0,73 | 0,05 |
| **ie180** | 0,58 | -0,41 | 0,58 | 0,85 |  | **ie180** | 0,21 | -0,04 | 0,51 | 0,53 |
| **us6** | 0,01 | 0,00 | 0,03 | 0,16 |  | **us6** | 0,04 | 0,02 | 0,42 | 0,54 |
| **us9** | 0,02 | 0,03 | 0,18 | 0,76 |  | **us9** | 0,07 | 0,07 | 0,79 | 0,35 |
| **ul51** | 0,07 | -0,01 | 3,10 | 9,09 |  | **ul51** | 0,03 | 0,80 | 0,74 | -0,36 |
| **ul49,5** | 0,00 | 0,02 | 0,20 | 0,55 |  | **ul49,5** | 0,07 | 0,02 | 0,57 | 0,40 |
| **ul48** | 0,03 | 0,09 | 0,22 | 0,83 |  | **ul48** | 0,06 | 0,01 | 0,71 | 0,40 |
| **ul32** | 0,02 | 0,11 | 1,12 | 2,29 |  | **ul32** | 0,08 | 0,12 | 1,94 | -0,54 |
| **ul33** | 0,07 | -0,01 | 0,64 | 1,22 |  | **ul33** | 0,06 | 0,02 | 0,48 | 0,56 |
| **ul36** | 0,17 | 0,11 | -0,03 | 0,64 |  | **ul36** | 0,29 | -0,17 | 0,48 | 0,40 |
| **ul37** | 0,01 | 0,03 | 0,22 | 1,36 |  | **ul37** | 0,03 | 0,03 | 0,68 | 0,30 |
| **ul38** | 0,06 | 0,19 | -0,07 | 1,73 |  | **ul38** | 0,08 | 0,05 | 0,99 | -0,05 |
| **ul41** | 0,01 | 0,08 | 0,52 | 0,83 |  | **ul41** | 0,21 | 0,05 | 0,71 | 0,18 |
| **ul42** | 0,01 | 0,05 | 0,52 | 0,72 |  | **ul42** | 0,04 | 0,10 | 0,92 | -0,06 |
| **ul44** | 0,00 | 0,00 | 0,04 | 0,20 |  | **ul44** | 0,00 | 0,01 | 0,53 | 0,52 |
| **ul24** | 0,01 | 0,00 | 0,09 | 0,65 |  | **ul24** | 0,03 | 0,00 | 0,92 | 0,08 |
| **ul22** | 0,01 | 0,02 | 0,22 | 0,96 |  | **ul22** | 0,02 | 0,05 | 0,29 | 0,76 |
| **ul19** | 0,00 | 0,02 | 0,17 | 0,61 |  | **ul19** | 0,07 | 0,02 | 0,75 | 0,57 |
| **ul17** | 0,00 | 0,00 | 0,18 | 0,81 |  | **ul17** | 0,02 | 0,06 | 0,97 | 0,00 |
| **ul10** | 0,01 | 0,00 | 0,15 | 0,91 |  | **ul10** | 0,02 | 0,04 | 0,63 | 0,31 |
| **ul6** | 0,00 | 0,04 | 0,31 | 1,15 |  | **ul6** | 0,05 | 0,77 | 0,04 | 0,35 |
| **ul5** | 0,00 | 0,01 | 0,05 | 0,17 |  | **ul5** | 0,24 | 0,98 | 0,97 | -1,63 |
| **ul1** | 0,12 | 0,03 | 1,35 | 8,61 |  | **ul1** | 0,06 | 0,06 | 0,85 | 0,17 |
| **us1** | 0,02 | 0,05 | 0,04 | 0,86 |  | **us1** | 0,12 | -0,01 | 1,26 | 0,36 |
| **LAT** | 0,19 | 0,82 | -0,87 | 1,05 |  | **LAT** | 0,10 | 0,09 | 1,13 | -0,26 |

**c**. The R_a_ values (R_(t+1)_/R_t_)

|  | **Low MOI** | | |  |  | **High MOI** | | |
| --- | --- | --- | --- | --- | --- | --- | --- | --- |
|  | **2h/1h** | **4h/2h** | **6h/4h** |  |  | **2h/1h** | **4h/2h** | **6h/4h** |
| **ul54** | 2,44 | 3,97 | 1,75 |  | **ul54** | 0,83 | 3,61 | 1,13 |
| **ul52** | 4,00 | 9,69 | 2,17 |  | **ul52** | 1,10 | 1,76 | 1,02 |
| **ul50** | 3,84 | 3,83 | 1,48 |  | **ul50** | 0,67 | 3,81 | 0,86 |
| **ul29** | 2,03 | 2,74 | 1,12 |  | **ul29** | 1,92 | 1,66 | 0,84 |
| **ul30** | 1,52 | 0,98 | 2,02 |  | **ul30** | 1,46 | 1,36 | 0,80 |
| **ul23** | 1,81 | 5,58 | 1,25 |  | **ul23** | 2,26 | 3,23 | 0,66 |
| **ul21** | 1,77 | 8,00 | 1,57 |  | **ul21** | 1,70 | 6,67 | 1,25 |
| **ul9** | 3,48 | 2,47 | 1,84 |  | **ul9** | 1,50 | 2,49 | 1,15 |
| **ep0** | 1,91 | 4,69 | 1,21 |  | **ep0** | 1,10 | 1,42 | 0,47 |
| **us3** | 2,48 | 3,36 | 0,87 |  | **us3** | 1,98 | 1,87 | 0,79 |
| **AST** | 2,83 | 0,02 | 14,91 |  | **AST** | 1,05 | 11,29 | 1,97 |
| **ul43** | 6,01 | 2,78 | 1,49 |  | **ul43** | 2,05 | 4,92 | 1,06 |
| **ul20** | 2,10 | 11,41 | 4,62 |  | **ul20** | 2,36 | 3,34 | 1,57 |
| **ul15** | 7,43 | 7,79 | 3,11 |  | **ul15** | 2,77 | 3,03 | 1,51 |
| **ul14** | 2,76 | 4,32 | 2,05 |  | **ul14** | 1,71 | 3,80 | 1,05 |
| **ie180** | 0,28 | 4,56 | 2,13 |  | **ie180** | 0,79 | 4,13 | 1,80 |
| **us6** | 1,66 | 4,76 | 4,75 |  | **us6** | 1,36 | 8,40 | 2,14 |
| **us9** | 3,15 | 4,70 | 4,28 |  | **us9** | 1,99 | 6,60 | 1,37 |
| **ul51** | 0,78 | 62,24 | 3,88 |  | **ul51** | 24,97 | 1,89 | 0,77 |
| **ul49,5** | 15,06 | 9,27 | 3,43 |  | **ul49,5** | 1,26 | 7,83 | 1,62 |
| **ul48** | 3,98 | 2,83 | 3,44 |  | **ul48** | 1,21 | 10,29 | 1,50 |
| **ul32** | 6,62 | 9,64 | 2,83 |  | **ul32** | 2,54 | 11,10 | 0,74 |
| **ul33** | 0,85 | 11,07 | 2,74 |  | **ul33** | 1,29 | 7,34 | 2,01 |
| **ul36** | 1,66 | 0,90 | 3,50 |  | **ul36** | 0,41 | 5,03 | 1,68 |
| **ul37** | 6,93 | 6,35 | 6,32 |  | **ul37** | 1,90 | 11,55 | 1,40 |
| **ul38** | 4,35 | 0,73 | 10,47 |  | **ul38** | 1,65 | 8,88 | 0,95 |
| **ul41** | 8,20 | 6,84 | 2,37 |  | **ul41** | 1,25 | 3,74 | 1,18 |
| **ul42** | 5,21 | 8,99 | 2,22 |  | **ul42** | 3,38 | 7,33 | 0,95 |
| **ul44** | 0,74 | 96,67 | 5,76 |  | **ul44** | 4,01 | 30,14 | 1,94 |
| **ul24** | 1,21 | 14,05 | 7,78 |  | **ul24** | 0,97 | 35,21 | 1,09 |
| **ul22** | 2,85 | 9,37 | 4,84 |  | **ul22** | 3,38 | 4,81 | 3,10 |
| **ul19** | 5,15 | 8,58 | 4,17 |  | **ul19** | 1,27 | 10,11 | 1,68 |
| **ul17** | 1,09 | 88,17 | 5,46 |  | **ul17** | 3,69 | 12,06 | 1,00 |
| **ul10** | 0,79 | 21,38 | 6,98 |  | **ul10** | 2,76 | 11,44 | 1,45 |
| **ul6** | 11,64 | 7,89 | 4,27 |  | **ul6** | 15,69 | 1,05 | 1,41 |
| **ul5** | 3,80 | 4,28 | 3,52 |  | **ul5** | 5,06 | 1,80 | 0,26 |
| **ul1** | 1,22 | 10,41 | 6,77 |  | **ul1** | 2,08 | 8,01 | 1,17 |
| **us1** | 3,55 | 1,52 | 9,20 |  | **us1** | 0,88 | 13,32 | 1,26 |
| **LAT** | 5,35 | 0,14 | 8,19 |  | **LAT** | 1,97 | 6,99 | 0,81 |
