# Supplementary material for: The effects of viral load on pseudorabies virus gene expression
Source: BMC Microbiol. 2010 Dec 6;10:311. doi: 10.1186/1471-2180-10-311 (PMC3016322; doi:10.1186/1471-2180-10-311)
Supplement: Additional file 3 — Comparison of R, RΔ and Ra values of low and high MOI infection by Pearson correlation. [file 1471-2180-10-311-S3.DOC]

Additional file 3. Comparison of R, R_Δ_ and R_a_ values of low and high MOI infection by Pearson correlation.

| **gene** | **R** |  | **gene** | **R_∆_** |  | **gene** | **Ra** |
| --- | --- | --- | --- | --- | --- | --- | --- |
| **ul54** | 0,95 |  | **ul54** | 0,47 |  | **ul54** | 0,92 |
| **ul52** | 0,89 |  | **ul52** | -0,35 |  | **ul52** | 0,99 |
| **ul50** | 0,86 |  | **ul50** | 0,74 |  | **ul50** | 0,45 |
| **ul29** | 0,91 |  | **ul29** | 0,97 |  | **ul29** | 0,77 |
| **ul30** | 0,43 |  | **ul30** | 0,32 |  | **ul30** | -0,78 |
| **ul23** | 0,85 |  | **ul23** | -0,09 |  | **ul23** | 0,85 |
| **ul21** | 0,99 |  | **ul21** | 0,54 |  | **ul21** | 1,00 |
| **ul9** | 0,95 |  | **ul9** | -0,20 |  | **ul9** | 0,12 |
| **ep0** | 0,02 |  | **ep0** | -0,67 |  | **ep0** | 0,87 |
| **us3** | 0,97 |  | **us3** | 0,87 |  | **us3** | 0,91 |
| **AST** | -0,57 |  | **AST** | -0,68 |  | **AST** | -0,58 |
| **ul43** | 0,96 |  | **ul43** | -0,03 |  | **ul43** | 0,01 |
| **ul20** | 0,92 |  | **ul20** | 0,65 |  | **ul20** | 0,75 |
| **ul15** | 0,95 |  | **ul15** | 0,65 |  | **ul15** | 1,00 |
| **ul14** | 0,90 |  | **ul14** | -0,07 |  | **ul14** | 1,00 |
| **ie180** | 0,95 |  | **ie180** | 0,59 |  | **ie180** | 0,99 |
| **us6** | 0,96 |  | **us6** | -0,22 |  | **us6** | 0,59 |
| **us9** | 0,87 |  | **us9** | 0,21 |  | **us9** | 0,63 |
| **ul51** | 0,50 |  | **ul51** | -0,67 |  | **ul51** | -0,50 |
| **ul49,5** | 0,95 |  | **ul49,5** | -0,06 |  | **ul49,5** | -0,05 |
| **ul48** | 0,91 |  | **ul48** | 0,37 |  | **ul48** | -0,90 |
| **ul32** | 0,68 |  | **ul32** | -0,23 |  | **ul32** | 0,91 |
| **ul33** | 0,99 |  | **ul33** | 0,90 |  | **ul33** | 1,00 |
| **ul36** | 0,76 |  | **ul36** | -0,18 |  | **ul36** | -0,52 |
| **ul37** | 0,84 |  | **ul37** | 0,22 |  | **ul37** | -0,43 |
| **ul38** | 0,56 |  | **ul38** | -0,56 |  | **ul38** | -0,83 |
| **ul41** | 0,92 |  | **ul41** | 0,31 |  | **ul41** | 0,32 |
| **ul42** | 0,85 |  | **ul42** | 1,00 |  | **ul42** | 1,00 |
| **ul44** | 0,94 |  | **ul44** | -0,28 |  | **ul44** | 0,99 |
| **ul24** | 0,71 |  | **ul24** | 0,55 |  | **ul24** | 0,86 |
| **ul22** | 0,99 |  | **ul22** | 0,99 |  | **ul22** | 0,90 |
| **ul19** | 0,94 |  | **ul19** | 0,90 |  | **ul19** | 0,97 |
| **ul17** | 0,72 |  | **ul17** | -0,15 |  | **ul17** | 0,96 |
| **ul10** | 0,85 |  | **ul10** | 0,88 |  | **ul10** | 0,91 |
| **ul6** | 0,74 |  | **ul6** | -0,08 |  | **ul6** | 0,86 |
| **ul5** | -0,14 |  | **ul5** | 0,97 |  | **ul5** | 0,16 |
| **ul1** | 0,76 |  | **ul1** | -0,08 |  | **ul1** | 0,72 |
| **us1** | 0,75 |  | **us1** | -0,08 |  | **us1** | -0,69 |
| **LAT** | -0,02 |  | **LAT** | -0,95 |  | **LAT** | -0,98 |
